# Spasm of the Neck of the Uterus

**Published:** 1897-03

**Authors:** 


Spasm of the Neck of the Uterus. M. Strebel, after a long series of observations, thinks that spasm of the neck of the uterus is very rare, if it really exists at all. By this term he means where there is a persistent contraction of the neck of the uterus lasting two, three, four, or even five days. With all due respect to the various veterinarians who have written on the subject, he thinks it is only a natural condition, and that spasm of the neck of the uterus is only an indication that the contractions are premature, and when the proper time has arrived the neck will relax and labor will proceed in a normal way. He thinks surgical interference should only be made when the neck of the uterus is found indurated, due to calcareous infiltration, and that the various therapeutic methods employed at present, such as belladonna, atropine, injections of hot water, etc., enjoy a reputation they do not deserve. In the majority of cases he advises that the animal should be let alone, awaiting developments.—*Schweizer Archives.*

